# Barriers and gaps in headache education: a national cross-sectional survey of neurology residents in Denmark

**DOI:** 10.1186/s12909-022-03299-6

**Published:** 2022-04-01

**Authors:** Thien Phu Do, Mikala Dømgaard, Simon Stefansen, Espen Saxhaug Kristoffersen, Messoud Ashina, Jakob Møller Hansen

**Affiliations:** 1 Danish Headache Center, Department of Neurology, Faculty of Health and Medical Sciences, Rigshospitalet Glostrup, University of Copenhagen, Copenhagen, Denmark; 2 Danish Knowledge Center On Headache Disorders, Rigshospitalet Glostrup, Valdemar Hansens Vej 5, 2600 Glostrup, Denmark; 3 Department of Neurology, Akershus University Hospital, Lørenskog, Norway

**Keywords:** Training, Education, Barrier, Residency, Headache, Migraine, Medication overuse, Tension-type headache

## Abstract

**Background:**

A major barrier to adequate headache care is the relative lack of formal education and training of healthcare professionals. Concerted efforts should be made to pinpoint major gaps in knowledge in healthcare professionals to facilitate better educational policies in headache training. The aim of this study was to identify deficiencies and barriers in headache training among residents in neurology in Denmark.

**Methods:**

We conducted a national cross-sectional survey of residents in neurology in Denmark from April 2019 to September 2019. The survey included questions on participant demographics, knowledge of and barriers in headache disorders, guidelines and diagnostic tools usage, contact with primary and tertiary care, medication overuse, and non-pharmacological interventions. Furthermore, respondents were asked to provide a ranked list from most to least interesting for six sub-specializations/disorders, i.e., cerebrovascular disease, dementia, epilepsy, headache, multiple sclerosis, Parkinson's disease.

**Results:**

Sixty (40%) out of estimated a population of ~ 150 resident across Denmark accepted the invitation. Of these, 54/60 (90%) completed the survey. Although two-thirds, 35/54 (65%), of the respondents had prior formalized training in headache disorders, we identified gaps in all explored domains including diagnosis, management, and referral patterns. Particularly, there was an inconsistent use of guidelines and diagnostic criteria from the Danish Headache Society (2.74 (± 1.14)), the Danish Neurological Society (3.15 (± 0.86)), and the International Classification of Headache Disorders (2.33 (± 1.08)); 1: never/have not heard of, 4: always. Headache was ranked second to last out of six sub-specializations in interest.

**Conclusions:**

Overall knowledge on headache disorders amongst neurology residents in Denmark do not meet the expectations set out by national and international recommendations. Stakeholders should make strategic initiatives for structured education in headache for improved clinical outcomes in parallel with costs reduction through resource optimization.

**Supplementary Information:**

The online version contains supplementary material available at 10.1186/s12909-022-03299-6.

## Introduction

Headache disorders are leading contributors to years lived with disability worldwide [1]. This is a largely avoidable addition to global disease burden since cost-effective treatments exist for the largest contributors, i.e., migraine and tension-type headache [2–5]. Despite this reality, serious deficiencies are reported worldwide in awareness among healthcare providers [2]. Indeed, the largest barriers to adequate headache care are found in the relative lack of formal education and training of healthcare professionals in wealthy nations and low- and middle-income countries (LMICs) alike [2, 6]. Typically, worldwide, only four hours on average are dedicated to headache disorders in undergraduate medical curricula, and a similar picture is found in postgraduate neurology specialization [6]. Furthermore, limited funding within the field of headache research continues to be a barrier [7].

In Denmark, headache disorders make up more than one-third of all disability-adjusted life years (DALYs) due to neurological disorders according to the *Global Burden of Disease *[8]. Neurology specialist training is largely categorized into the introduction program (1^st^ year residents) and the main program (2^nd^, 3^rd^, 4^th^, and 5^th^year residents). It is expected that residents at all levels obtain knowledge about headache disorders through clinical experience in combination with self-study, but there is no mandatory formalized course in headache until residents are enrolled into the main program. Furthermore, while there is a requirement of experience with headache management obtained through specialist outpatient clinics, there is no formalized requirement of a dedicated training rotation in headache with a set amount of hours. These factors allow for a discrepancy in knowledge during the fundamental years of future neurologists [9]. These challenges are not limited to Denmark, but also extends to other regions including the United States [10, 11]. Concerted efforts should be made to pinpoint major gaps in knowledge in healthcare professionals to facilitate better educational policies in headache training. The aim of this study was to identify deficiencies and barriers in headache training among residents in neurology in Denmark.

## Methods

### Overview

The present study is a national cross-sectional survey of self-reported knowledge of residents in neurology in Denmark conducted from April 2019 to September 2019. Protocols for conducting of surveys is subject to exemption from processing at the National Committee on Health Research Ethics in Denmark. The ethical approval for this study was exempted by the National Committee on Health Research Ethics in Denmark. All methods were carried out in accordance with the Declaration of Helsinki. Informed consent was obtained from all subjects and/or their legal guardian(s). We handled survey data confidentially and maintained anonymity of respondents throughout the study.

### Questionnaire

The survey was designed by clinicians and experts in headache disorders from the Danish Headache Center and Akershus University Hospital in collaboration with the Danish Knowledge Center on Headache Disorders, a non-profit organization focusing on raising the level of knowledge about headaches both among professionals and patients. The survey included questions related to participant demographics, knowledge of and barriers in headache disorders, guideline and diagnostic tools usage. Furthermore, we included topics of particular interest includingmedication overuse and non-pharmacological interventions. A full overview of questions is provided in Supplemental File 1.

### Surveys

Surveys were sent to the residency training directors and departmental chairs of all neurological departments for distribution among their current residents. Furthermore, contacts were asked about the number of residents at their department. Of note, neurology specialist training is largely categorized into the introduction program (1^st^ year residents) and the main program (2^nd^, 3^rd^, 4^th^, and 5^th^ year residents). Pediatric neurology is not included in this survey as we only invited residents in a neurological residency; in Denmark, pediatric neurology is a sub-specialization of pediatrics. We did not conduct a pilot trial prior to the survey. The initial invitation was sent out in April 2019. Reminders were sent after two weeks to the training directors.

### Statistical analysis

We performed descriptive analyses of the data in Microsoft Excel, version 2103 (16.0.13901.20400) / April 13, 2021. We present data as frequencies or means with standard deviations (SD).

## Results

### Demographics

**Fig. 1:**
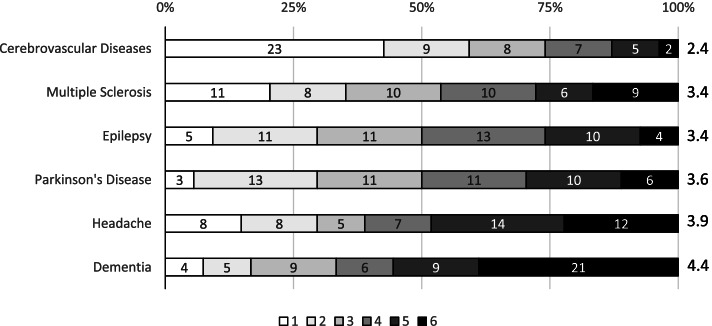
Interest in neurological sub-specializations. All respondents were asked to list neurological sub-specializations in a ranked order, 1: most interesting, 6: least interesting. The number represent how many respondents ranked the sub-specialization a specific rank, e.g., 23 respondents ranked “Cerebrovascular Diseases” at the top of their list, i.e., rank 1. “Cerebrovascular diseases” is on average ranked as the most interesting sub-specialization whereas”Headache” ranks second to last. 1: highest ranked, 6; lowest ranked

We identified 15 neurological departments in Denmark across five regions; of these, 14/15 departments were included as one was excluded due to no current residents associated with the department. There is no official tally of number of residents in Denmark, but we estimated a population of ~ 150 residents based on information derived from residency training directors. Sixty residents from the 14 included departments accepted the invitation, which corresponds to 40% of all possible potential participants; 54/60 (90%) of respondents completed all questions of the survey. Participants were from all five regions of Denmark with an approximate even distribution between residents in the introduction program and main program (Table 1); 35 of respondents had previously participated in a headache education program or training (Table 1). Headache disorders ranked as the second to least popular sub-specialization among residents (Fig. 1).

**Table 1:** Respondent demographics

**Participants**	54
**Region**	
● Capital Region of Denmark	21 (39%)
● Central Denmark Region	5 (9%)
● North Denmark Region	8 (15%)
● Region of Southern Denmark	12 (22%)
● Region Zealand	8 (15%)
**Residency program**	
● Introductory program	25 (46%)
● Main program	29 (54%)
**Prior headache education/training**	
● All residents	35 (65%)
○ Residents in introductory program	11 (44%)
○ Residents in main program	24 (83%)

A total of 54 participants across all five regions in Denmark were included in the survey. There was an approximately even distribution between residents currently enrolled in the introduction program (1^st^ year residents) and the main program (2^nd^, 3^rd^, 4^th^ and 5^th^ year residents). Two-thirds had prior training in headache disorders

### Knowledge, barriers and challenges

Residents were most knowledgeable about tension-type headache and migraine whereas knowledge on post-traumatic headache was reported as most lacking (Table 2). The common disease-oriented barriers were challenging diagnostics, unclear medical history, and lack of effective treatment options. The largest physician-oriented barrier was reported as lack of prescribed efficacy of given treatment.

**Table 2:** Self-reported knowledge, barriers, and challenges in headache disorders

	**All participants** (*n* = 54)	**Introduction program** (*n* = 25)	**Main program** (*n* = 29)
**Self-reported knowledge of headache disorders**^**a**^	**Mean (SD)**	**Mean (SD)**	**Mean (SD)**
● Migraine	3.91 (0.65)	3.76 (0.83)	4.03 (0.42)
● Tension-type headache	3.94 (0.65)	3.80 (0.82)	4.07 (0.46)
● Cluster headache	3.68 (0.75)	3.33 (0.92)	3.89 (0.49)
● Trigeminal neuralgia	3.48 (0.84)	3.12 (0.93)	3.79 (0.62)
● Medication overuse headache	3.56 (0.86)	3.28 (0.89)	3.39 (0.77)
● Post-traumatic headache	3.15 (0.97)	2.96 (0.98)	3.31 (0.97)
**Patient and disease-oriented barriers**^**b**^	**n (%)**	**n (%)**	**n (%)**
● Challenging diagnosis	31 (57%)	15 (60%)	16 (55%)
● Comorbidities	23 (43%)	9 (36%)	14 (48%)
● Patient anxiety of adverse events	13 (23%)	4 (16%)	9 (31%)
● Treatment failure due to adverse events	19 (35%)	6 (24%)	13 (45%)
● Unclear medical history	31 (57%)	17 (68%)	14 (48%)
● Lack of effective treatment options	31 (57%)	12 (48%)	19 (66%)
● No challenges	3 (6%)	1 (4%)	2 (7%)
● Other	8 (15%)	3 (12%)	5 (17%)
**Physician-oriented barriers**^**b**^	**n (%)**	**n (%)**	**n (%)**
● Own lack of knowledge	17 (31%)	12 (48%)	5 (17%)
● Find headache patients difficult to diagnose and treat	26 (48%)	12 (48%)	14 (48%)
● Challenges in physician/patient collaboration	17 (31%)	6 (24%)	11 (38%)
● Insufficient consultation time	15 (28%)	3 (12%)	12 (41%)
● Lack of prescribed treatment efficacy	31 (57%)	16 (64%)	15 (52%)
● Insufficient support from other specialists	13 (24%)	2 (8%)	11 (38%)
● No barriers	5 (9%)	3 (12%)	2 (7%)

^a^Scale from 1–5; 1: very bad, 5: very good. ^b^Participants were asked to choose one or more options

### Guidelines and diagnostic tools

The majority of respondents reported that they use guidelines for management of headache disorders (Table 3). Overall respondents rank an inconsistent use of the International Classification of Headache Disorders (ICHD) [12] and headache diaries for diagnosis (2.33 (1.08), 3.31 (0.77), respectively; 1: never/have not heard of, 4: always). For outcome assessment, residents rate a higher consistent use of headache diaries and quality of life parameters (3.14 (0.93), 3.04 (0.80), respectively; 1: never/have not heard of, 4: always).

**Table 3:** Use of guidelines, classification and tools for diagnosis and outcome assessment

	**All participants** (*n* = 54)	**Introduction program** (*n* = 25)	**Main program** (*n* = 29)
**Guidelines**	**Mean (SD)**	**Mean (SD)**	**Mean (SD)**
● Guidelines from the Danish Headache Society	2.74 (1.14)	2.4 (1.19)	3.03 (1.02)
● Guidelines from the Danish Neurological Society	3.15 (0.86)	2.96 (1.02)	3.31 (0.66)
**Diagnostic tools**	**Mean (SD)**	**Mean (SD)**	**Mean (SD)**
● The International Classification of Headache Disorders	2.33 (1.08)	2.36 (1.04)	2.31 (1.14)
● Headache diary for diagnosis	3.31 (0.77)	3.28 (0.84)	3.34 (0.72)
**Outcome assessment**	**Mean (SD)**	**Mean (SD)**	**Mean (SD)**
● Headache calendar for outcome assessment	3.14 (0.93)	2.96 (1.09)	3.31 (0.76)
● Quality of life parameters (e.g., sickness absence, reduced participation in social events)	3.04 (0.80)	2.88 (0.89)	3.17 (0.71)

Scale from 1–4; 1: never/have not heard of, 4: always

### Contact and referral patterns

In most cases, the respondents estimate that headache consultations take up 11–20% of patient contacts (Table 4). Contact and collaboration with primary care is inconsistent and is skewered towards a lower score (2.37 (1.29), 2.63 (0.79), respectively; 1: never/very bad, 5: very frequently). Residents largely estimate 11–20% of consultations need referral to a tertiary center. The most common reason for further referral to tertiary/specialist care was lack of treatment efficacy and diagnostic uncertainty. When asked about whether residents find it beneficial to refer patients to tertiary care, the result was skewed towards a negative outcome (not at all), and one-fifth reported they did not know.

**Table 4:** Contact and referral patterns

	**All participants** (*n* = 54)	**Introduction program** (*n* = 25)	**Main program** (*n* = 29)
**Proportion of consultations related to headache**	**n (%)**	**n (%)**	**n (%)**
● 1–10%	13 (24%)	10 (40%)	3 (10%)
● 11–20%	29 (54%)	7 (28%)	22 (76%)
● 21–30%	8 (15%)	5 (20%)	3 (10%)
● 31–40%	0 (0%)	0 (0%)	0 (0%)
● > 40%	4 (7%)	3 (12%)	1 (3%)
● None	0 (0%)	0 (0%)	0 (0%)
**Primary care**	**Mean (SD)**	**Mean (SD)**	**Mean (SD)**
● Contact from primary care for professional advice on headache^a^	2.37 (1.29)	1.89 (0.96)	2.86 (1.36)
● Collaboration with primary care for referred headache patients^b^	2.63 (0.79)	2.60 (0.77)	2.66 (0.83)
**Proportion of headache patients referred to tertiary/specialist care**	**n (%)**	**n (%)**	**n (%)**
● 1–10%	9 (17%)	4 (16%)	5 (17%)
● 11–20%	35 (65%)	14 (56%)	21 (72%)
● 21–30%	7 (13%)	5 (20%)	2 (7%)
● 31–40%	1 (2%)	1 (4%)	0 (0%)
● > 40%	1 (2%)	0 (0%)	1 (3%)
● Never	1 (2%)	1 (4%)	0 (0%)
**Most common reason for referring to tertiary/specialist care**^**c**^	**n (%)**	**n (%)**	**n (%)**
• Diagnostic uncertainty	20 (37%)	11 (44%)	9 (31%)
• Suspicion of serious underlying cause	2 (4%)	2 (8%)	0 (0%)
• Lack of treatment efficacy	32 (59%)	11 (44%)	21 (72%)
• Desire/expectation of the patient	11 (20%)	5 (20%)	6 (21%)
•Other	8 (15%)	5 (20%)	3 (10%)
**Wait time for referral to tertiary/specialist care**	**n (%)**	**n (%)**	**n (%)**
● Short	0 (0%)	0 (0%)	0 (0%)
● Acceptable	16 (30%)	11 (44%)	5 (17%)
● Long	21 (39%)	8 (32%)	13 (45%)
● Unacceptably long	8 (15%)	2 (8%)	6 (21%)
● Do not know	9 (17%)	4 (16%)	5 (21%)
**Helpful for patients to be referred to tertiary/specialist care**^**d**^	**Mean (SD)** 2.33 (0.74)	**Mean (SD)** 2.00 (0.76)	**Mean (SD)** 2.62 (0.74)

^a^Scale from 1–5; 1: never, 5: very frequently. ^b^Scale from 1–5; 1: none/very bad, 5: very good. ^c^Participants could choose up to two answers. ^d^Scale from 1–5; 1: not at all, 5: to a great extent. 10 (19%) responded they did not know

### Medication overuse

Participants on average ranked that medication overuse is a problem during clinical management of headache disorders (Table 5). The majority of respondents could correctly identify simple analgesics and migraine acute medications as potential causes of medication overuse headache whereas fewer could correctly identify opioids. The majority (80%) could provide the recommended maximum use of simple analgesics.

**Table 5:** Medication overuse headache

	**All participants** (*n* = 54)	**Introduction program** (*n* = 25)	**Main program** (*n* = 29)
**Medication overuse headache is a problem among your headache patients**^**a**^	M**ean (SD)** 3.31 (0.86)	**Mean (SD)** 3.04 (0.84)	**Mean (SD)** 3.55 (0.82)
**Kind of medications that can cause medication overuse headache**	**n (%)**	**n (%)**	**n (%)**
● Simple analgesics	53 (98%)	24 (83%)	29 (100%)
● Opioids	32 (59%)	12 (48%)	20 (69%)
● Migraine acute medicine (e.g., triptans)	42 (78%)	16 (64%)	26 (90%)
● Migraine preventive medicine (e.g., beta blockers)	5 (9%)	2 (8%)	3 (10%)
● Do not know	0 (0%)	0 (0%)	0 (0%)
**Recommended maximum use of simple analgesics for headache patients**	**n (%)**	**n (%)**	**n (%)**
● 1 day a week	1 (2%)	1 (4%)	0 (0%)
● 2–3 days a week	43 (80%)	17 (68%)	26 (90%)
● 4–5 days a week	4 (7%)	1 (4%)	3 (10%)
● 6 days a week	0 (0%)	0 (0%)	0 (0%)
● Do not know	6 (11%)	6 (24%)	0 (0%)

^a^Scale from 1–5; 1: not at all, 5: to a great extent

### Non-pharmacological interventions

Respondents were neutral (mean: 3 (1.13); 1: never, 5: very frequently) whether patients seek advice on non-pharmacological treatments. When asked about whether they feel equipped for this task, the result was skewered towards not at all (Table 6). The most popular recommended non-pharmacological interventions were physiotherapy, exercise, and psychological treatment.

**Table 6:** Non-pharmacological interventions

	**All participants** (*n* = 54)	**Introduction program** (*n* = 25)	**Main program** (*n* = 29)
**Use of non-pharmacological interventions**	**Mean (SD)**	**Mean (SD)**	**Mean (SD)**
● Patients seek advice on non-pharmacological treatment options^a^	3.00 (1.13)	2.84 (1.25)	3.14 (1.03)
● Feel equipped to advise patients on non-pharmacological treatment options^b^	2.29 (0.82)	2.08 (0.81)	2.48 (0.76)
**Recommended non-pharmacological interventions**^**c**^	**n (%)**	**n (%)**	**n (%)**
● Acupuncture	15 (28%)	9 (36%)	6 (24%)
● Craniosacral therapy	3 (6%)	1 (4%)	2 (7%)
● Diet	28 (52%)	13 (52%)	15 (52%)
● Ear (daith) piercing	1 (2%)	0 (0%)	1 (3%)
● Exercise	45 (83%)	19 (76%)	26 (90%)
● Medical cannabis	0 (0%)	0 (0%)	0 (0%)
● Neurostimulation	1 (2%)	1 (4%)	0 (0%)
● Physiotherapy	50 (93%)	22 (88%)	28 (97%)
● Psychological treatment	41 (76%)	17 (68%)	24 (83%)
● Reflexology	4 (7%)	2 (8%)	2 (7%)
● Other	6 (11%)	3 (12%)	3 (10%)
● None of the above	0 (0%)	0 (0%)	0 (0%)

^a^Scale from 1–5; 1: never, 5: very frequently. ^b^Scale from 1–5; 1: not at all, 5: to a great extent. ^c^Participants could choose multiple answers

## Discussion

In this national cross-sectional survey of neurology residents in Denmark, we identified several areas for improvement for headache education of healthcare providers.

### Diagnostic criteria

Approximate half of respondents report that diagnosis and treatment of patients with headache is challenging (Table 2). This is particularly worrying as the majority estimate 11–20% of consultations are related to headache (Table 4) – a significant proportion. Yet these findings are not surprising and in line with findings from studies conducted in other regions [13–15]. As there are no biomarkers or diagnostic tests for most headache disorders, diagnosis rely on the medical history. While headache diaries in general are used for diagnosis and outcome assessment, there is an apparent inconsistent use of the ICHD (Table 3). The expectation is that residents would have a more consistent use and knowledge of diagnostic criteria over time due to accumulation of experience, but the pattern is similar for residents in the introduction program and the main program. With a limited number of training hours during residency, the low utilization of specific diagnostic criteria may be caused by a higher emphasis on stratification of cases into high-risk (secondary headache disorders, e.g., headache attributed to trauma) and low-risk patients (primary headache disorders, e.g., migraine) rather than specific diagnoses during early training [16–18]. This is not unreasonable as secondary headache disorders may cause significant morbidity, and for some etiologies, a relatively high mortality [17]. Nonetheless, correct diagnosis is the mainstay of clinical management of primary headache disorders, and targeted educational interventions are needed. In an international survey of neurologists, explicit diagnostic criteria are only used in 56% of cases [6]. These data confirm that these deficiencies are not necessarily corrected after completion of specialization and substantiates the need for improvement already during residency.

### Treatment and management

More than half of respondents identify lack of treatment options and efficacy as a barrier to care (Table 2). While it cannot be excluded that this is due to rare headache disorders with few evidence-based options, cost-effective treatments do exist for the largest headache burdens, i.e., migraine and tension-type headache [2–5]. These findings are surprising as most residents are more than moderately confident in their self-reported knowledge of headache disorders (Table 2). Furthermore, less than two-thirds of respondents could correctly identify opioids as a potential cause of medication overuse headache, and more worrying, a few respondents both in the introduction program and main program incorrectly reported prophylactic medications as a potential cause (Table 5). This misinterpretation can lead to a worse clinical outcome, and provides a possible explanation of poor use of preventive medications in eligible cases [19]. Most respondents recommended one non-pharmacological interventions (Table 6), which provides a multidisciplinary approach to clinical management. However, for some of the more popular recommended options (e.g., physiotherapy, acupuncture, and diet), data on potential therapeutic gain of these therapies is discordant, and may also explain why respondents on average feel less confident in advising patients on non-pharmacological treatment options [2]. These gaps in treatment and management could be related to a lacking use of available national guidelines [20], but the use of guidelines is reported to be higher than moderate (Table 3).

### Primary care and tertiary care

In Denmark, headache services are divided in three levels: primary care (general practitioner), specialist care (general neurology), and tertiary care (specialized headache center). Headache is the most common neurological symptom in primary care [21], and should in 90% of cases be initiated and maintained in primary care [22, 23]. While there are cases where specialist care can be necessary, treatment of a headache patient and repatriation to primary care should be coordinated with the general practitioner to ensure continuity of care. However, contact from primary care for professional advice on headache and collaboration with primary care for referred headache patients is inadequate in the present study (Table 4). A possible consequence is unnecessary escalation and referral to tertiary care. This is also reflected by the fact that most respondents estimated up to one-fifth of patients require referral to tertiary care with one of the common reasons being diagnostic uncertainty (Table 4), which may be caused by the inconsistent use of diagnostic criteria provided by ICHD (Table 3). For migraine, presumably compromising the largest proportion of patients, requires only 1% of cases to be referred to tertiary care [2]. Specialist services are scarce and impeded by long waiting lists [2]. This is also the case in a high-income country as Denmark, where more than half of the residents estimated the waiting list to be either long or unacceptable. Furthermore, while tertiary care do provide better care due to greater expertise and access to a multidisciplinary approach [24], residents do not necessarily find it beneficial for patients to be referred (Table 4).

### Barriers to care

The most common patient and disease-related barriers were connected to diagnosis and treatment (Table 2). An unclear medical history is reported by more than half of residents as an impediment, which may also overlap with comorbidities also being reported as a common barrier [2, 4, 25]. This is troubling as diagnosis of headache disorders rely on the medical history. Interestingly, these may be related to a high frequency of challenges in physician/patient collaboration and insufficient consultation time as both would affect obtaining a good medical history.

### Headache education

Even if headache training is not mandatory until the main program, almost half of all residents in the introduction program had already completed a formalized course in headache prior to this survey (Table 1). This likely reflects an interest and need for education already at an early career stage. *The European Union of Medical Specialists*categorize applied clinical knowledge in four different levels, and it is recommended that trainees obtain at level 3 and 4 within the first two years of training [26]; level 4 is the ability to make a complete diagnosis and optimize treatment. As such, residents in the main program should be confident in all aspects of headache management before completion of specialization, however, not all residents had completed formalized headache training prior to the survey, and there were gaps in all explored domains. Almost one-fifth of residents in the main program reported their own knowledge as a personal barrier to care (Table 2). Overall, the expectations are discordant with the actual level of self-reported knowledge. In a survey of neurology chairs and resident directors in the United States, two-thirds of respondents found headache education inadequate or had no opinion [11]. Implementation of a mandatory rotation in an specialized outpatient clinic with a set number of hours improved gaps in an United States-based institution [27], and it is not unreasonable to assume that a similar intervention may improve the findings of our survey. While this cannot be concluded based on the available data, one may speculate whether the overall low interest in headache as a sub-specialization is an important factor (Fig. 1) [28, 29]. Increased availability and emergence of novel disease-specific treatment options and scientific advances may help improve interest in the future [3, 30–32].

### Strengths and limitations

This is the first national cross-sectional study of residents in neurology in Denmark. The study included approximate 40% of all residents in Denmark, which we evaluate as representative of the population as the sample included residents from both inside and outside the Capital Region of Denmark (greater Copenhagen area). Nonetheless, as there is no official tally of number of residents in Denmark, and that we included less than half the possible the estimated number of residents, we may have introduced a selection bias. Furthermore, we did not inquire about specific year of training for residents in the main program, which spans from 2^nd^ to 5^th^ year residents. Surveys may introduce recall bias, but we find no suspect systematic bias in this domain.

## Conclusions

Even in a developed country such as Denmark with excellent headache services [33], the overall knowledge of neurology residents on headache disorders do not meet the expectations set out by both national and international recommendations. We identified several deficiencies and barriers in headache management amongst residents particularly related to diagnosis. Parallel investigations should be investigated at other levels of systems of care (e.g., primary care) and in other regions to assess for similar trends. Strategic initiatives for structured education in headache would likely result in improved clinical outcomes in parallel with costs reductions and should be prioritized by both regional and national stakeholders.

## Supplementary Information

**Additional file 1.**

## Data Availability

Anonymized datasets generated and/or analyzed during the current study are available from the corresponding author upon reasonable request and following the acquisition of necessary permissions.

## Abbreviations

DALY: Disability-adjusted life year
ICHD: International Classification of Headache Disorders
LMIC: Low- and middle-income country
