# Supplementary material for: Barriers and gaps in headache education: a national cross-sectional survey of neurology residents in Denmark
Source: BMC Med Educ. 2022 Apr 1;22:233. doi: 10.1186/s12909-022-03299-6 (PMC8976293; doi:10.1186/s12909-022-03299-6)
Supplement: Supplementary file 1 — Additional file 1. [file 12909_2022_3299_MOESM1_ESM.docx]

**Supplemental File 1.** Overview of survey questions for “Barriers and Gaps in Headache Education: A National Cross-sectional Survey of Neurology Residents in Denmark “

Self-Reported Knowledge, Barriers, and Challenges in Headache Disorders

How would you rate your knowledge of:

|  | Very bad |  |  |  | Very good |
| --- | --- | --- | --- | --- | --- |
| Migraine | ❑ | ❑ | ❑ | ❑ | ❑ |
| Tension type headache | ❑ | ❑ | ❑ | ❑ | ❑ |
| Cluster Headache (Hortons) | ❑ | ❑ | ❑ | ❑ | ❑ |
| Trigeminal neuralgia | ❑ | ❑ | ❑ | ❑ | ❑ |
| Medication overuse headache (MOH) | ❑ | ❑ | ❑ | ❑ | ❑ |
| Posttraumatic headache | ❑ | ❑ | ❑ | ❑ | ❑ |

**Do you experience any of the following challenges in relation to your headache patients?**

❑ Difficult to diagnose

❑ Comorbidity makes treatment difficult

❑ Not possible to initiate treatment due to patient fear of side effects

❑ Failure to comply with initiated treatment due to side effects

❑ Unclear medical history

❑ Lack of effective treatment options

❑ No challenges

❑ Other – describe _____

**What do you see as the biggest barriers to optimal treatment of your headache patients?**

| ❑ My lack of knowledge  ❑ Headache patients are difficult to diagnose and treat  ❑ Challenges in collaboration between patient and therapist  ❑ Insufficient time for the patient  ❑ Lack of treatment effect  ❑ Insufficient support from / collaboration with other specialists  ❑ I experience no barriers |
| --- |

Use of Guidelines, Classification and Tools for Diagnosis and Outcome Assessment
When diagnosing and treating headache, how often:

|  | Never / have never heard of |  |  | Always |  |
| --- | --- | --- | --- | --- | --- |
| Do you use the guidelines from the Danish Headache Society? | ❑ | ❑ | ❑ | ❑ |  |
| Do you use the guidelines from the Danish Neurological Society? | ❑ | ❑ | ❑ | ❑ |  |
| Do you use the International Classification of Headache Disorders (ICHD)? | ❑ | ❑ | ❑ | ❑ |  |
| Do you ask patients to complete a headache diary to make a diagnosis? | ❑ | ❑ | ❑ | ❑ |  |
| Do you use a headache calendar for follow-up on treatment? | ❑ | ❑ | ❑ | ❑ |  |

Do you routinely ask your patients if their headache affects their quality of life? (e.g. reduced participation in social events, sickness absence)?

❑ Never

❑ Less than half of the time

❑ At least half of the time

❑ Always

**Contact and Referral Patterns**

What proportion of your patient consultations are related to headache?

❑ 1-10%

❑ 11-20%

❑ 21-30%

❑ 31-40%

❑ >40%

❑ None

Do general practitioners contact you for professional advice regarding headache patients?

❑ Never

❑ Rarely

❑ Once in a while

❑ Frequently

❑ Very frequently

**How would you describe your collaboration with General Practitioners concerning referred headache patients?**

❑ Very bad/Non existing

❑ Bad

❑ Neither good nor bad

❑ Good

❑ Very good

❑ Non existing

What proportion of your headache patients do you refer to treatment at specialized headache centers?

❑ 1-10%

❑ 11-20%

❑ 21-30%

❑ 31-40%

❑ >40%

❑ I never refer patients

What is your most common reason for referring headache patients?

❑ Diagnostic uncertainty

❑ Suspicion of serious underlying cause

❑ Lack of treatment effect

❑ Desire/expectation of the patient

❑ Other reason, please describe _____

**How do you consider the wait time for patients you refer to specialized treatment?**

❑ Short

❑ Acceptable

❑ Long

❑ Unacceptable long

**To what extent do you find it helpful for patients to be referred?**

❑ Not at all

❑ To a small extent

❑ To some extent

❑ To a large extent

❑ To a great extent

❑ Do not know

**Medication Overuse Headache**

**To what extent do you find that medication overuse headache is a problem among your headache patients?**

❑ Not at all

❑ To a small extent

❑ To some extent

❑ To a large extent

❑ To a great extent

**Do you know what kind of medication that can potentially cause medication overuse headache?**

❑ Simple analgesics

❑ Opioids

❑ Migraine acute medicine (e.g. triptans)

❑ Migraine preventive medicine (e.g. beta blockers)

❑ Do not know

**Do you know the recommended maximum use of simple analgesics for headache patients (to avoid medication overuse headaches)?**

| ❑ 1 day a week  ❑ 2-3 days a week  ❑ 4-5 days a week  ❑ 6 days a week  ❑ Do not know |
| --- |

**Non-Pharmacological Interventions**

**Does your patients seek your advice on non-pharmacological treatment options for headaches (officially recognized as well as complementary and alternative options)?**

❑ Never

❑ Rarely

❑ Once in a while

❑ Frequently

❑ Very frequently

**To what extent do you feel equipped to advise your patients on non-medical treatment options?**

❑ Not at all

❑ To a small extent

❑ To some extent

❑ To a large extent

❑ To a great extent

**What types of treatment would you recommend if asked by your patients?**

❑ Physiotherapy

❑ Psychological treatment

❑ Medical cannabis

❑ Acupuncture

❑ Reflexology

❑ Diet

❑ Exercise

❑ Cranio sacral therapy

❑ Neurostimulation

❑ Ear piercing

❑ I do not recommend any of these treatments

**Do you recommend other types of treatment?**________________________________________________________________________________

Interest in Neurological Sub-specializations

Rank the following - most interesting as number 1

|  | 1 | 2 | 3 | 4 | 5 | 6 |
| --- | --- | --- | --- | --- | --- | --- |
| Cerebrovascular diseases | ❑ | ❑ | ❑ | ❑ | ❑ | ❑ |
| Dementia | ❑ | ❑ | ❑ | ❑ | ❑ | ❑ |
| Epilepsy | ❑ | ❑ | ❑ | ❑ | ❑ | ❑ |
| Headache | ❑ | ❑ | ❑ | ❑ | ❑ | ❑ |
| Multiple sclerosis | ❑ | ❑ | ❑ | ❑ | ❑ | ❑ |
| Parkinson’s | ❑ | ❑ | ❑ | ❑ | ❑ | ❑ |
